# Ocular Adipostructuring in Complications From Orbital Fillers: Managing the Orbital Base

**DOI:** 10.7759/cureus.115512

**Published:** 2026-08-31

**Authors:** Manuel Sequera, Gladys Velazco, Macarena Olivares, Eduvigis Solorzano

**Affiliations:** 1 Medical Academy, Instituto Chileno de Rejuvenecimiento y Optimización en Medicina Estética, Santiago, CHL; 2 Facial Harmonization, Centro Latinoamericano de Entrenamiento Médico e investigación, Bogota, COL; 3 Medical Academy, Icrom Medical Santiago de Chile, Santiago, CHL; 4 Dentistry, Universidad de Los Andes, Mérida, VEN

**Keywords:** adipostructuring, biomaterials, canulas, complicaciones perioculares, hematomas, orbital fillers, periocular complications

## Abstract

The periocular contour is one of the most complex and vulnerable anatomical regions of the face and is highly prone to complications following the injection of biomaterials such as liquid polycaprolactone (PCL), hyaluronic acid (HA), and polylactic acid (PLA). These complications include persistent hyperpigmentation, malar edema, fibrosis, sensation of pressure, structural irregularities, and lymphatic dysfunction. Periocular adipostructuring is a specialized variant of facial adipostructuring. The technique uses blunt-tipped cannulas (25G and 27G × 50 mm), precise maneuvers for controlled tissue dissection, and localized delivery of the active components of the FaceStructure® Kit (Mioface Harmony, Cúcuta, Colombia), together with adjuvant agents such as *Melilotus officinalis* and organic silicon. Through this approach, fibrosis is released, mechanotransduction is activated, the clearance of ferric pigments is promoted, and lymphatic drainage is stimulated, thereby restoring the anatomical architecture without inducing additional volume or trauma. This is a retrospective experimental study in which 30 patients presenting with clinical complications of the periocular region secondary to the placement of PCL, PLA, and HA fillers were treated, distributed as follows: PCL = 12; PLA = 10; and HA = 8. The study included 22 females and eight males aged between 35 and 60 years. The results show that, despite involving complications in the periocular contour, most patients achieved clinical recovery, and the treatment was associated with a histological shift consistent with the remodeling of localized fibrosis into a more organized and functional dermal tissue. Using this technique in complicated patients has allowed the development of a functional regenerative intervention model focused on restoring optimal anatomical conditions so that the tissue can recover autonomously, providing a reproducible, safe solution that is applicable to a broad spectrum of complications.

## Introduction

The periorbital area is one of the most delicate and functionally complex anatomical regions of the human face. Its three-dimensional structure and layered composition make this area a real challenge, both aesthetically and clinically. Aging, genetic factors, postural changes, and environmental influences affect the eye contour early on, producing visible signs like volume loss, changes in pigmentation, hollowing, protrusion of fat pads, and alterations in skin quality [1]. In response to these manifestations, contemporary aesthetic medicine has incorporated a wide range of treatments with biomaterials, such as dermal fillers and biostimulators, including hyaluronic acid, calcium hydroxylapatite, and more recently, liquid polycaprolactone (PCL) [2].

Although these resources have shown satisfactory results in other areas of the face, their use around the eyes has historically been considered high-risk. The dense vascular network, the superficial lymphatic system, the proximity to the eyeball, and the high neurosensory sensitivity mean that any improper intervention can trigger significant functional and aesthetic complications [3]. Among the most common complications are persistent bruising, prolonged cheek swelling, reactive fibrosis, secondary hyperpigmentation, a feeling of heaviness or pressure, and, in some cases, occlusive necrosis [4].

A pigmentary interaction has been observed between extravasated blood and the PCL, which causes a persistent grayish or brown discoloration that does not respond to conventional treatments and is often misdiagnosed as dermal or melanic hyperpigmentation [5]. These complications not only change the patient's appearance, but also affect their emotional well-being, harm the doctor-patient relationship, and expose the professional to ethical, legal, and reputational issues [6].

In this context, periorbital adipostructuring emerges as an advanced adipostructuring technique applied specifically to the eye contour. Its goal is not to fill, but to reorganize deep and superficial tissues through the mechanical and biological activation of anatomical planes, without resorting to suction or the volumetric deposition of synthetic substances [7]. In this way, it positions itself as a tool for anatomical, functional, and aesthetic reorganization that respects the physiology of the periorbital region, stimulates tissue regeneration, and allows for the non-invasive resolution of complications caused by previously injected biomaterials.

It is based on the use of blunt cannulas 25G and 27G, 50 mm, with specific anatomical dissection maneuvers and the sequential administration of active ingredients that are senolytic, anti-inflammatory, draining, and biostimulating, included in the FaceStructure® Kit (Mioface Harmony, Cúcuta, Colombia), including *Centella asiatica*, organic silicon, melilot extract, carnitine, dimethylaminoethanol (DMAE), and pyruvic acid [8]. The protocol aims to activate mechanotransduction mechanisms, promote the tissue elimination of hemosiderin and residual metabolites, stimulate deep lymphatic drainage, and restore the anatomical structure without causing fibrosis or excessive volume [9].

The eye contour area includes a complex anatomical region made up of multiple layers that have structural, neuromuscular, vascular, lymphatic, and expressive functions. A detailed understanding of this architecture is essential for any aesthetic or regenerative approach in the area, especially when dealing with complications from procedures involving biomaterials. Each layer is arranged in a precise way and is closely interconnected with the others, so any change can cause morphofunctional imbalances with a significant clinical impact [10].

The skin around the eyes is the thinnest in the human body, with an average thickness of about 0.5 mm, lacks a true hypodermis, and shows minimal sebaceous activity [11]. These characteristics make it extremely vulnerable to dehydration, post-inflammatory pigmentation, loss of elasticity, and the cumulative effects of photophysical damage. The delicate network of superficial capillaries that supplies this area also contributes to the development of persistent bruising when leakage occurs as a result of trauma or inflammation.

Located just beneath the skin, the orbicularis oculi muscle of the periorbital region is a striated sphincter-type muscle that surrounds the orbit and allows the eyelids to close. Anatomically, it is divided into three parts: pretarsal, preseptal, and orbital [12]. Dysfunctional contraction or fibrosis secondary to exogenous materials can impair lymphatic drainage and lead to malar edema or altered facial expression.

The periorbital contour is supported by a network of retaining ligaments that define discrete fat compartments and stabilize the overlying soft tissues [13]. When these ligaments are compromised by fibrosis or poorly positioned biomaterials, they can lose their support function, which can lead to tissue sagging, loss of structural support, and a deeper infraorbital groove.

Periocular fat tissue is organized into superficial and deep fat pads. Inferiorly, three main compartments are commonly identified: medial, central, and lateral [14]. These structures are dynamic and physiologically mobile. Their prominence or displacement related to aging, trauma, or inappropriate cosmetic procedures can distort facial contours, creating a pseudohernia, deep grooves, or a double contour effect. Displaced materials in this area tend to cause compression, fibrosis, and chronic inflammation.

The orbit is bordered by a bony ring made up of the maxilla, the zygomatic bone, the frontal bone, and the sphenoid. The infraorbital rim is a key spot for ligament attachment and an important landmark for both clinical and surgical procedures [15]. As we age, progressive bone resorption contributes to infraorbital hollowing and a downward shift of the surrounding soft tissues. Filler treatments that ignore this skeletal remodeling can be ineffective or even harmful, as they try to mask a structural deficit without restoring the underlying bone support.

The periorbital area is innervated by branches of the trigeminal nerve (cranial nerve V), primarily the infraorbital nerve, a branch of the maxillary division, as well as branches of the facial nerve (cranial nerve VII) that control facial muscle expression [16]. Changes caused by chemical inflammation, fibrosis, or mechanical compression can lead to paresthesia, a feeling of pressure, or even chronic dysesthesia. Therefore, a technique that respects neurosensory anatomy is essential to preserve both sensory and motor function.

The periorbital lymphatic system consists of a dense and fragile network of superficial vessels that mainly drain into the preauricular and submandibular lymph nodes [17]. The physiologically slow nature of this drainage means that any inflammatory or mechanical overload tends to show up as persistent swelling. When biomaterials that induce fibrosis or pigmentation, like PCL, are injected, lymphatic flow can get blocked, preventing the removal of hemosiderin and other cellular debris. This blockage keeps the tear duct looking pigmented, congested, or puffy.

Based on our experience with 30 clinical cases successfully managed with this technique, we have observed not only aesthetic resolution of the complication but also functional restoration of the lymphatic system and the reorganization of the deep ligament framework. In this article, we present a detailed anatomical review of the eye contour, clinical foundations of periorbital adipostructuring, and a standardized protocol to address complications arising from the improper use of biomaterials in this highly sensitive and functionally critical area.

## Materials and methods

The periorbital area comprises a complex anatomical region consisting of multiple layers that have structural, neuromuscular, vascular, lymphatic, and expressive functions, as shown in Figure 1.

**Figure 1:**
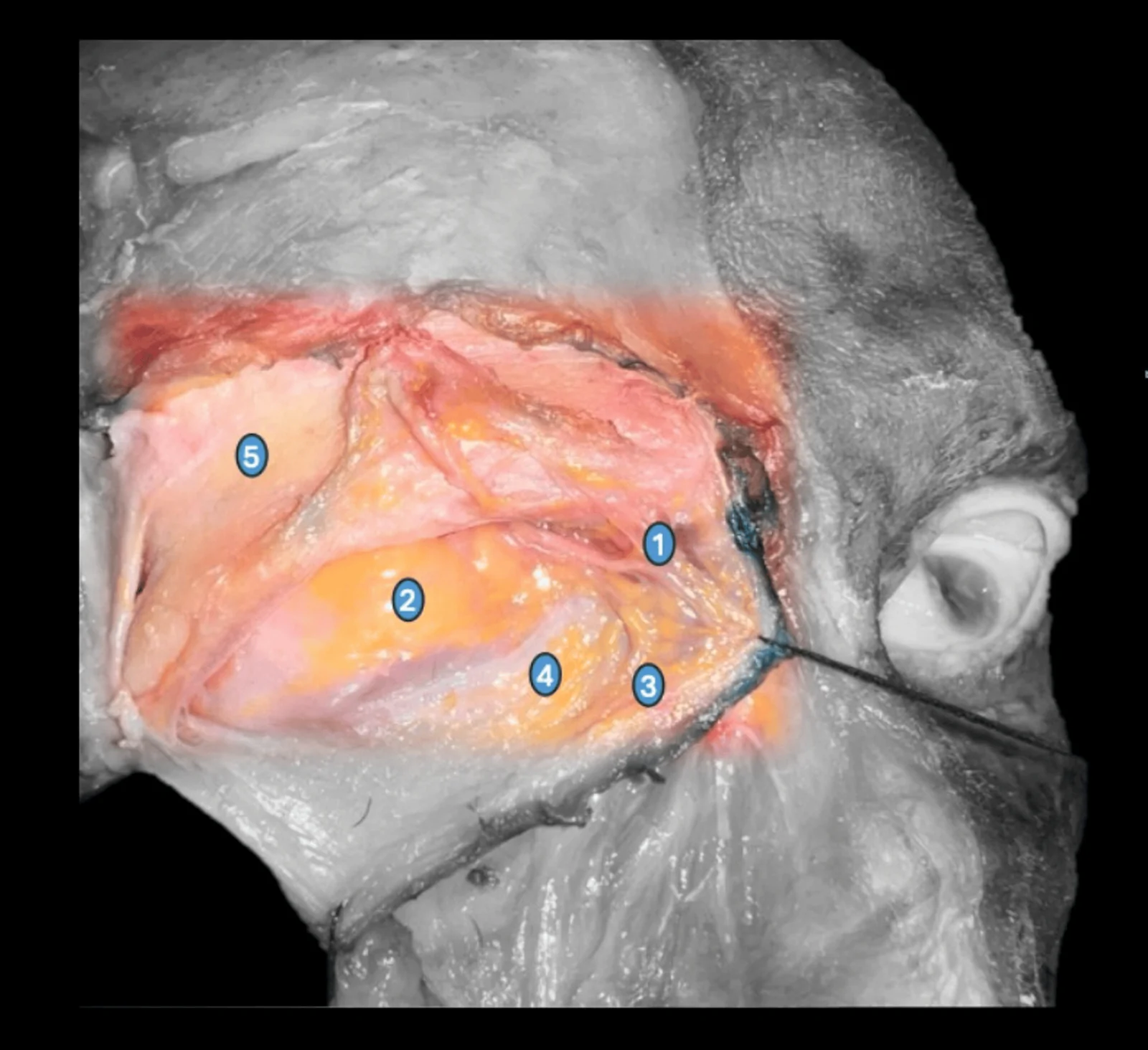
Deep dissection of the periorbital contour showing (1) Whitnall’s ligament, (2) inferior retro-orbital fat, (3) superficial fat and orbicularis oculi fascia, (4) sub-orbicularis oculi fat (SOOF) at its superior border and bony attachment, and (5) supraorbital periosteum.

This was a retrospective, experimental study that included 30 patients diagnosed with clinical complications of the periorbital region following the injection of PCL, poly‑L‑lactic acid (PLLA), or hyaluronic acid (HA). The cohort was distributed as follows: 12 cases related to PCL, 10 to PLLA, and eight to HA, comprising 22 females and eight males between 35 and 60 years of age.

All patients were evaluated and diagnosed in consultation, and a detailed clinical history was obtained. At baseline, the main findings were distributed as follows: nine patients presented with motor alterations, and 21 showed persistent bruising and induration of the treated area.

The investigation adhered to the ethical principles of the Declaration of Helsinki; therefore, each patient provided written informed consent after receiving a full explanation of the nature and objectives of the treatment.

All patients were treated with the periorbital adipostructuring technique. This is an advanced procedure within regenerative aesthetic medicine, specifically designed to reorganize the tissues of the ocular contour without resorting to invasive surgery or the infiltration of synthetic biomaterials. Conceptually, it represents a specialized extension of the facial adipostructuring protocol, adapting its biomechanical and biological principles to the complex periocular anatomy [7]. Its purpose is not to fill or camouflage volume deficits, but to reorganize local anatomical architecture, restore the functionality of tissue compartments, and promote tissue regeneration through mechanical and bioactive stimuli. In addition, it integrates detailed anatomical knowledge, regenerative biomechanics, and applied biological pharmacology, in line with contemporary concepts of functional aesthetic medicine. It clearly differs from techniques based on filler deposition, cutting, suction, or thermal injury.

Anatomical marking and instrumentation

The procedure is performed exclusively with blunt cannulas of 25G and 27G, 50 mm in length, which allow navigation between anatomical planes without transecting structures or injuring superficial capillaries. Anatomical marking is carried out with the patient in a seated position, assessing facial symmetry, malar projection, the infraorbital groove, and the distribution of fat pads. Safe entry points are identified, generally via a lateral trans‑malar or infrazygomatic approach, while respecting areas of higher neurovascular density. During the procedure, the cannula is advanced along trajectories parallel to the orbital rim, delineating the medial, central, and lateral fat compartments, releasing adhesions, and promoting reorganization of the ligamentous system and deep connective tissue. The objective is to restore the deep supporting plane rather than to generate volume.

Active ingredients used

A key feature of the technique is the strategic co‑administration of active agents, selected according to the clinical diagnosis and the type of complication present. In cases with persistent hemosiderin‑related pigmentation, marked fibrosis, or evident lymphatic dysfunction, specific actives are used, such as *Melilotus officinalis* extract, with venotonic, lymphokinetic, and anti‑inflammatory properties, and organic silicon, which acts as a cofactor in collagen synthesis, connective tissue restructuring, and stabilization of the extracellular matrix. These agents are administered in micro‑focused doses within the compromised anatomical planes, delivered along the cannula track using progressive or retrograde deposition techniques, always under manual control and without applied pressure. Table 1 shows the material responsible for the complication and the active ingredient used to resolve it.

**Table 1:** Active ingredients used according to the type of complication. PCL: polycaprolactone; DMAE: dimethylaminoethanol.

Type of complication	Recommended active ingredients	Therapeutic objective
Persistent pigmentation due to hemosiderin and PCL	Melilotus officinalis extract, organic silicon	Lymphatic drainage, antioxidation, clearance of ferric pigments
Fibrosis or firm nodules	DMAE, organic silicon, arginine	Extracellular matrix remodeling, tissue relaxation
Persistent malar edema	Troxerutin, hesperidin, natural flavonoids	Veno‑lymphatic stimulation, anti‑inflammatory effect
Structural disharmony of the groove	Selective lipogenic agents from the FaceStructure® Kit	Regenerative activation of deep planes without adding volume
Paresthesia or sensation of pressure	Glutathione, vitamin B6, antioxidants	Neurosensory repair and reduction of neuroinflammation

The combination of active agents is adjusted according to the clinical presentation, using minimal, localized doses to avoid tissue saturation.

Before initiating the procedure, a comprehensive patient assessment is essential. This should include high‑resolution frontal photographic analysis; palpation of the area to identify zones of fibrosis, nodules, or irregularities; a gentle compression test to assess the degree of lymphatic stasis; evaluation of tissue mobility and cutaneous sensitivity; and a detailed history of previous procedures, including the type of material used, injection technique, and time elapsed since application. The information gathered allows classification of the complication according to its predominant origin (volumetric, pigmentary, fibrotic, neurosensory, lymphatic, or combined) and guides an individualized cannula‑based approach.

Preparation

Rigorous asepsis of the area with topical chlorhexidine and application of local anesthesia at the entry point (1% lidocaine without epinephrine).

Anatomical Access

A single entry point is used per hemiface, usually at the lateral third of the malar region, approximately 1.5 cm below the lateral orbital rim. The cannula is introduced in the submuscular plane, parallel to the infraorbital rim, advancing medially toward the inner canthus if required.

Controlled Non‑surgical Tissue Release

Slow in‑and‑out movements (fan-shaped maneuver) and micro‑rotations (axial torque) are performed to release adhesions, disrupt mild fibrosis, reposition fat compartments, and activate mechanotransduction. Superficial passes are avoided to prevent ecchymosis and dermal disruption.

Deposition of Active Ingredients

Once tissue release is completed, retrograde, micro‑dosed administration of the protocol’s active agents is performed, with emphasis on the most compromised zones. Administration is performed only after the avulsive cannula maneuver has been completed.

Post‑treatment and Follow‑Up

No massage or local compression is recommended after treatment. Patients should avoid direct sun exposure, intense heat, or vigorous physical exercise for 48 hours. High water intake and supportive draining nutricosmetics (e.g., *Centella* and rutin extracts) are advised. Clinical follow‑up is scheduled at 15 days, one month, and three months for photographic, clinical, and functional evaluation.

## Results

Of the 30 patients diagnosed with clinical complications in the periorbital area, recovery was evaluated in relation to the proportion of cases that did not recover. Figure 2 shows the ratio of resolved cases to unresolved cases.

**Figure 2:**
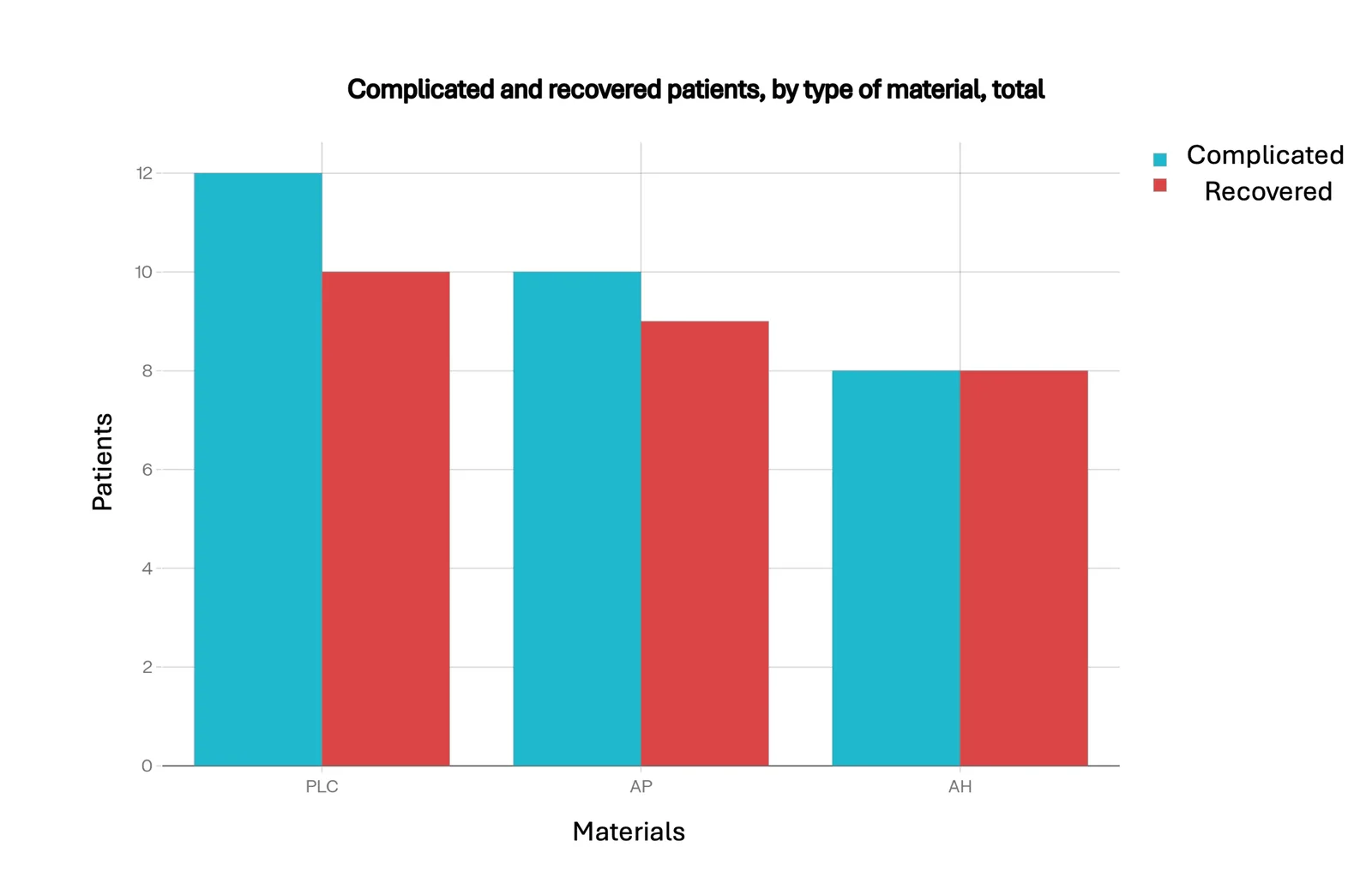
Bar chart showing the number of patients who presented complications in blue bars and the number of patients who achieved recovery after treatment in red bars. PLC: polycaprolactone; AP: polylactic acid; AH: hyaluronic acid.

The PCL group had 12 complicated cases out of 22 treated (12/22 ≈ 54.5%), with 10 recovered cases out of 22 (10/22 ≈ 45.5%). The PLLA group had 10 complicated cases out of 19 treated (10/19 ≈ 52.6%), with nine recovered cases out of 19 (9/19 ≈ 47.4%). The HA group had eight complicated cases out of 16 treated (8/16 = 50%), with eight recovered cases out of 16 (8/16 = 50%).

All patients underwent a biopsy before and after treatment to evaluate cellular changes histologically. Figure 3 shows the pre‑treatment biopsy samples.

**Figure 3:**
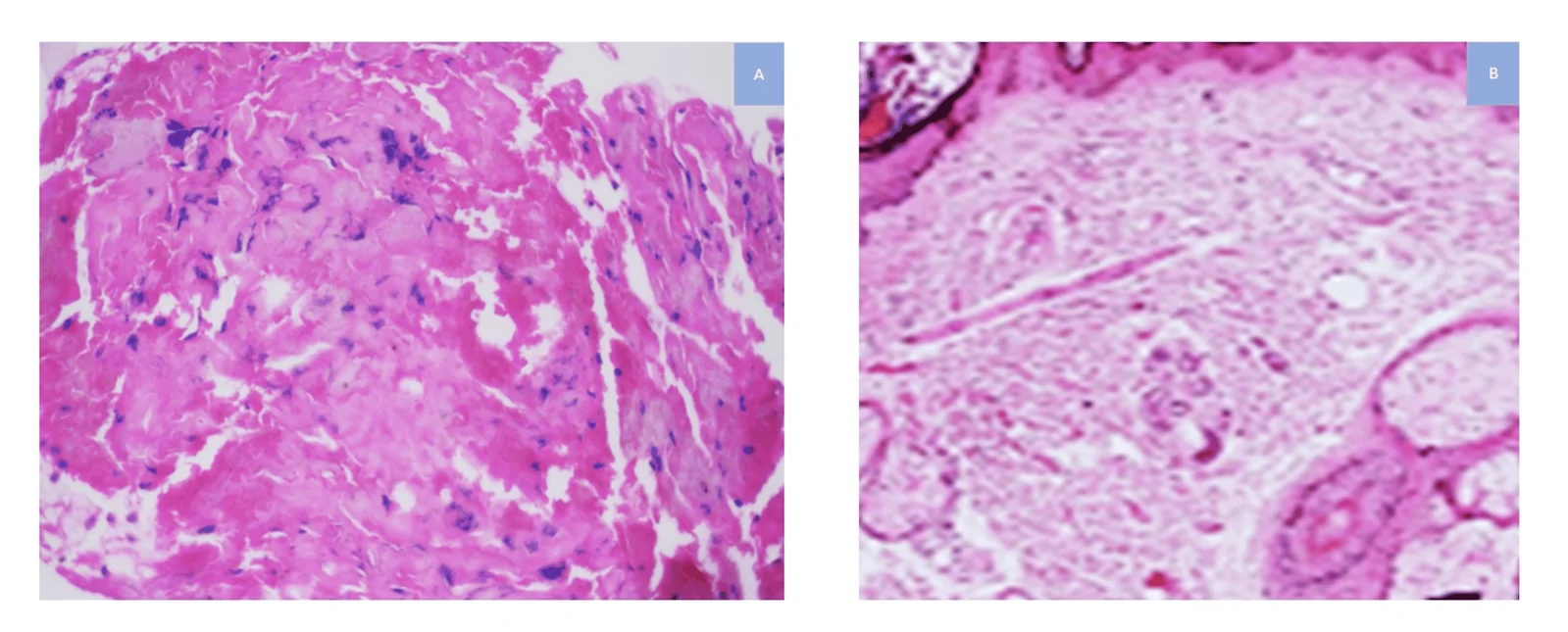
(A) Histological section stained with hematoxylin-eosin prior to treatment, showing a compact tissue pattern consistent with fibrosis or mature scar, characterized by densely packed collagen fibers. (B) Histological section stained with hematoxylin-eosin after treatment, showing tissue with a superficial epithelial band and a more eosinophilic, continuous zone suggestive of epidermis overlying a broad thickness of completely healthy dermis/connective tissue.

In Figure 3A, dense fibrous connective tissue is observed, with thick, wavy collagen bundles stained pink with hematoxylin-eosin (H&E). Between the collagen bundles, fibroblast nuclei and a few scattered spindle‑shaped cells can be seen. No significant inflammatory infiltrates are identified, although there are small, mild collections. At the margins, adipose tissue with preserved, normal architecture is evident. These findings are compatible with fibrosis or a mature scar, given the dense, compact arrangement of collagen fibers. The absence of marked inflammation supports a predominantly reparative or fibrotic process rather than an active inflammatory one. The adjacent adipose tissue shows no alterations, suggesting a localized process.

In Figure 3B, a superficial epithelial band is observed, with a more eosinophilic, continuous zone suggestive of an epidermis overlying a broad thickness of dermis/connective tissue. Beneath the dermis, multiple rounded, lobulated structures with a glandular or acinar appearance can be seen, consistent with preserved cutaneous adnexa (likely glandular or follicular tissue). There is a predominance of dense collagen in the dermis with a relatively organized architecture, which may correspond either to mature scar tissue or to normotrophic dermis, depending on the anatomical site and duration of the process. The preservation of adnexal structures and the absence of evident massive tissue destruction are favorable findings for biostimulation and adipostructuring protocols, as they indicate a relatively preserved connective and vascular scaffold, although this interpretation must be integrated with clinical data and additional stains (e.g., for elastic fibers, vascularization, and regeneration markers).

The patients’ subsequent photographs provided clinical evidence of the reported changes, demonstrating improvements in both aesthetics and function, as we can see in Figures 4-7.

**Figure 4:**
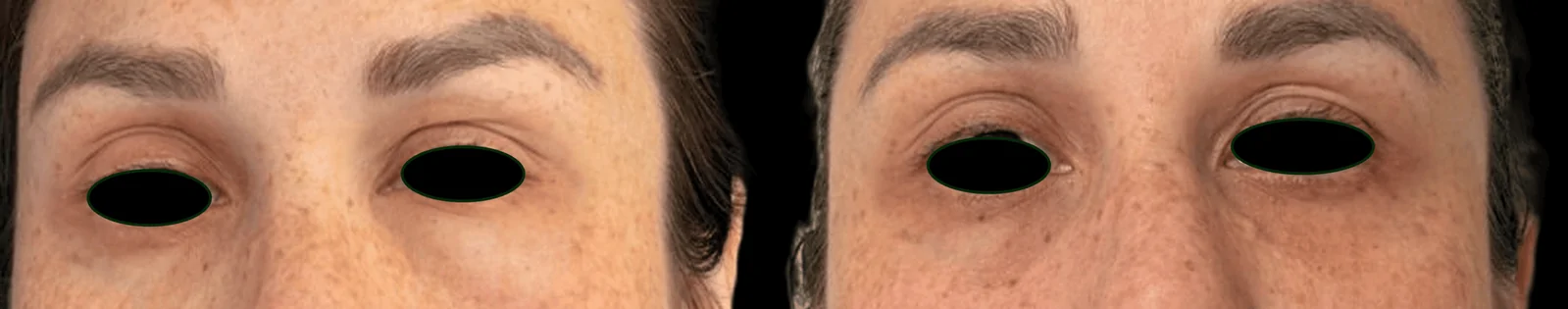
Patient before treatment, showing reduced palpebral opening due to induration in the treated area, and at three‑month follow‑up after treatment.

**Figure 5:**
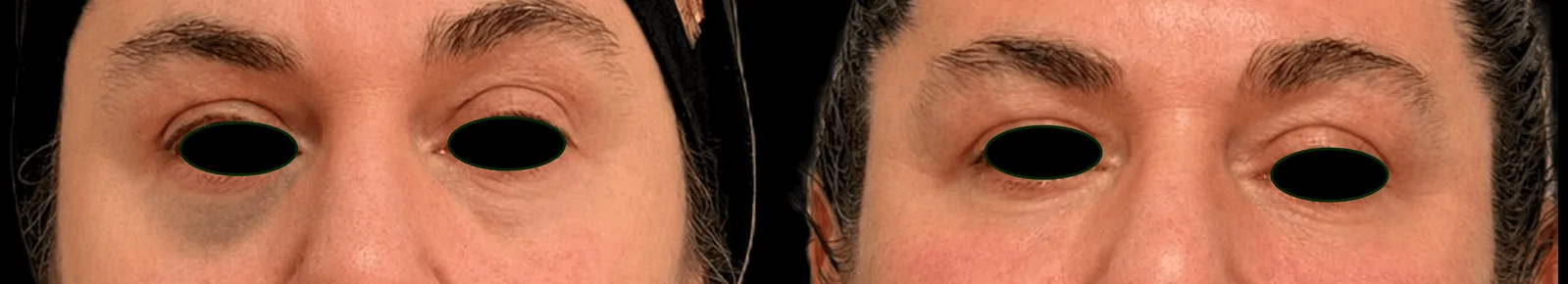
Persistent hematoma of 60 days’ duration and complete resolution of the associated pigmentation three months after treatment.

**Figure 6:**
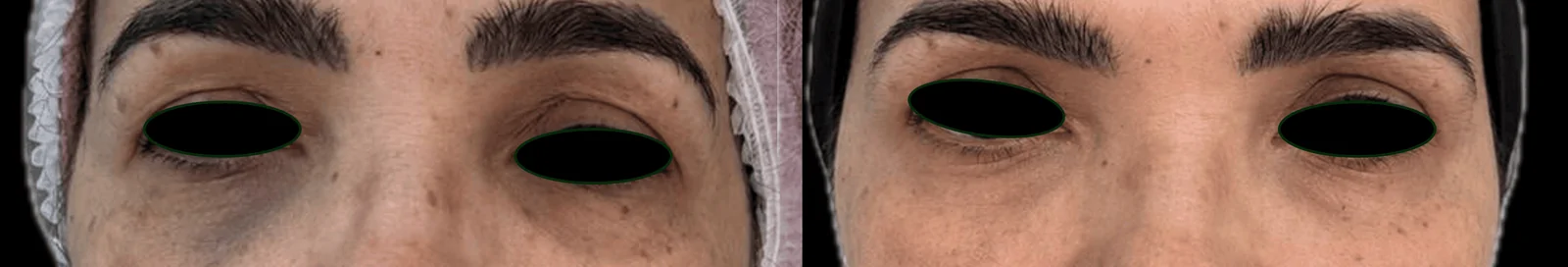
Persistent hematoma of 15 days’ duration and complete resolution of the associated pigmentation three months after treatment.

**Figure 7:**
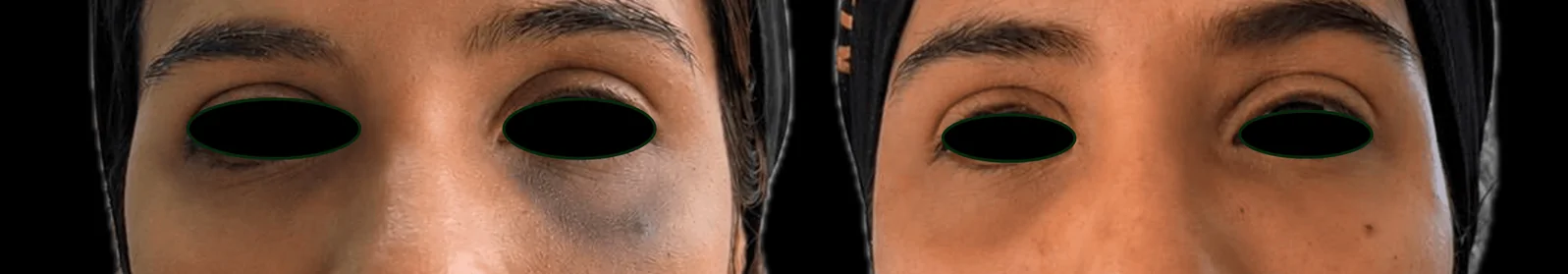
Persistent hematoma of 20 days’ duration and complete resolution of the associated pigmentation three months after treatment.

## Discussion

The findings of this case series demonstrate that, although all patients presented with complications affecting the periorbital region, the majority achieved favorable clinical recovery following treatment. Moreover, the intervention was associated with histological changes consistent with remodeling of localized fibrosis into a more organized and functionally restored dermal connective tissue.

Clinical recovery according to filler material

Among the 30 patients presenting with periocular complications, clinical recovery was documented in the majority of cases regardless of the filler material involved. As illustrated in Figure 2, the number of recovered patients closely approximated the number of complicated cases within each treatment group, indicating a consistently high overall response to the proposed therapeutic protocol.

The results summarized in Table 2 show that, in the PCL group, 12 of 22 patients (54.5%) developed complications, whereas 10 of 22 (45.5%) achieved clinical recovery, suggesting that nearly all complicated cases were successfully reversed. In the PLLA group, complications occurred in 10 of 19 patients (52.6%), while nine of 19 (47.4%) demonstrated complete clinical recovery. Likewise, in the HA group, eight of 16 patients (50%) developed complications, and all eight recovered completely, representing full resolution of complications within this subgroup.

Although the initial incidence of complications was comparable among the three biomaterials, the final clinical outcomes indicate that the proposed therapeutic protocol is capable of reversing adverse events even in the presence of persistent fibrosis and chronic ecchymosis, as observed throughout this series [18].

Baseline histological findings

Baseline biopsy specimens demonstrated dense fibrotic connective tissue composed of thick, compact collagen bundles with minimal inflammatory cellular infiltrate, a histopathological pattern consistent with localized fibrosis or mature scar tissue. These findings correspond to the classical histological features of cutaneous fibrosis, characterized by increased collagen synthesis, excessive extracellular matrix deposition, and fibroblast proliferation in the presence of minimal residual inflammatory activity [19].

The preservation of the adjacent adipose tissue architecture suggests that the fibrotic process remained localized rather than diffuse. This observation is particularly relevant for adipostructuring and regenerative biostimulation strategies, as it preserves a viable vascularized adipose substrate capable of supporting tissue remodeling.

Within the context of published reports describing periocular filler complications, which are typically characterized by edema, contour irregularities, persistent ecchymosis, and localized fibrosis rather than extensive tissue necrosis, these histological findings support the concept that the predominant pathological mechanism is one of aberrant tissue repair and remodeling rather than irreversible tissue destruction.

Histological findings following treatment

Post-treatment biopsy specimens revealed preservation of a continuous superficial epithelial layer consistent with an intact epidermis, together with a thicker dermis composed of healthy-appearing connective tissue characterized by dense yet more organized collagen bundles and preservation of the cutaneous adnexal structures.

This histological pattern is consistent with previous reports involving regenerative biostimulatory therapies and tissue-remodeling devices, in which reorganization of collagen fibers into finer and more orderly bundles has been associated with measurable improvements in skin texture, elasticity, and overall tissue quality [20,21].

Serial clinical photographs (Figures 4-7) documented both functional and aesthetic improvement. Patients presenting with eyelid induration demonstrated restoration of normal palpebral opening, while those with persistent ecchymosis achieved complete resolution of hematomas ranging from 15 to 60 days in duration. These observations are consistent with contemporary evidence indicating that most periocular filler-related complications are potentially reversible when managed through an integrated approach combining appropriate filler dissolution when indicated, hematoma management, lymphatic drainage, and tissue remodeling strategies [22].

The close correlation between the clinical disappearance of pigmentation and ecchymosis and the histological transition toward a more organized connective tissue architecture supports the hypothesis that the proposed treatment acts not only on residual biomaterial or organized hematoma, but also on the fibrotic extracellular matrix responsible for perpetuating structural and functional tissue alterations.

Taken together, the high proportion of patients achieving clinical recovery, the favorable histological remodeling observed after treatment, and the objective photographic evidence collectively support the effectiveness of the proposed protocol for the management of periorbital complications associated with PCL, PLLA, and HA. These findings suggest that the technique may represent a structured regenerative approach within current strategies for the prevention and management of adverse events in periocular aesthetic medicine [23].

Limitations of the study

This study has several limitations that should be considered when interpreting its results. First, this is a retrospective, uncontrolled case series without a comparison group (whether a conventional treatment group or an observation group), which prevents the establishment of a definitive causal relationship between the adipostructuring protocol and the observed resolution of hematomas, edema, and pigmentation. Nevertheless, the immediate clinical resolution observed following application of the technique is a verifiable and reproducible finding that supports a direct therapeutic effect. Second, the total sample size is small and unevenly distributed across the material subgroups, which limits the statistical power to detect true differences in recovery rates between PCL, PLLA, and HA. Third, clinical “recovery” was not defined using an objective, validated severity scale. Fourth, follow-up was limited to a maximum of three months, which is insufficient to assess the durability of the therapeutic effect or the possibility of late recurrence of fibrosis or pigmentation. Fifth, the histological evaluation was descriptive and qualitative (hematoxylin-eosin staining only), without quantitative histomorphometric analysis or additional stains/immunomarkers. Sixth, complications arising from three biomaterials with different degradation kinetics and interaction mechanisms (PCL, PLLA, HA) were grouped under a single treatment protocol and analyzed as if they were pathophysiologically equivalent, which could mask the true differences in treatment response among the materials. Prospective, multicenter, randomized studies with standardized outcome measures and longer follow-up are needed to corroborate these findings.

## Conclusions

Using this technique in complicated patients has allowed the development of a functional regenerative intervention model focused on restoring optimal anatomical conditions so that the tissue can recover by itself. This provides a reproducible, safe solution applicable to a wide range of biomaterial‑related complications and represents a potential turning point in how the medical community approaches the unintended consequences of poorly directed aesthetic treatments.

It is suggested that future research should include multicenter, collaborative, prospective studies with larger patient cohorts to systematize the outcomes of this technique, validate its regenerative effects through objective biomarkers, and integrate it into international clinical guidelines as a first‑line therapeutic option for the management of periocular complications. More than a technique, it represents a working philosophy based on respect for anatomy, understanding of tissue behavior, and trust in the biological processes of repair.
